# The Gazette and Dr. Holmes

**Published:** 1860-12

**Authors:** 


### THE GAZETTE AND DR. HOLMES.

*The American Medical Gazette* has periodical spasms at the state of the profession. Many years ago, the apparent severity of these attacks used to awaken our serious apprehensions, but constant experience in its methods for the ten or dozen years of its existence, has materially lessened our sympathizing alarms. We venture to prophecy that it will even recover from its attack of—Oliver Wendell Holmes.

The *Gazette*, evidently not having read the article, in a late number of this journal, of which it complains, makes au amusing blunder in its last issue.

The Massachusetts Medical College having rescinded the unmanly and impotent resolution which it first prefixed to the Prof.'s "Currents and Counter Currents," we can conceive now that the address stands before the profession solely on its merits, subject to endorsement either as a whole, or, like any other creation of mind, in part. Truth and error are very apt to be somewhat intermixed in this mundane sphere —we have even believed them at times, *rari nantes in gurgite vasto*, equally perceptible on the pages of the *Gazette*. As we expressed total dissent from Dr. Holmes in the proposition which the *Gazette* especially lashes, perhaps it may be induced on reconsideration of the subject not to "have an attack" of Dr. Brainard, for the literary, critical or professional sins of his correspondent, A.

